# A highly efficient atomically thin curved PdIr bimetallene electrocatalyst

**DOI:** 10.1093/nsr/nwab019

**Published:** 2021-02-02

**Authors:** Fan Lv, Bolong Huang, Jianrui Feng, Weiyu Zhang, Kai Wang, Na Li, Jinhui Zhou, Peng Zhou, Wenxiu Yang, Yaping Du, Dong Su, Shaojun Guo

**Affiliations:** School of Materials Science and Engineering, Peking University, Beijing 100871, China; Department of Applied Biology and Chemical Technology, Hong Kong Polytechnic University, Hong Kong, China; School of Materials Science and Engineering, Peking University, Beijing 100871, China; School of Materials Science and Engineering, Peking University, Beijing 100871, China; School of Materials Science and Engineering, Peking University, Beijing 100871, China; Frontier Institute of Science and Technology jointly with College of Science, Xi’an Jiaotong University, Xi’an 710054, China; Center for Functional Nanomaterials Brookhaven National Laboratory, Upton, NY 11973, USA; School of Materials Science and Engineering, Peking University, Beijing 100871, China; School of Materials Science and Engineering, Peking University, Beijing 100871, China; School of Materials Science and Engineering, Peking University, Beijing 100871, China; School of Materials Science and Engineering & National Institute for Advanced Materials, Nankai University, Tianjin 300350, China; Center for Functional Nanomaterials Brookhaven National Laboratory, Upton, NY 11973, USA; School of Materials Science and Engineering, Peking University, Beijing 100871, China; College of Engineering, Peking University, Beijing 100871, China

**Keywords:** strain, metallene, PdIr alloy, atomically thin, electrocatalyst

## Abstract

The multi-metallene with an ultrahigh surface area has great potential in precise tuning of surface heterogeneous d-electronic correlation by surface strain effect for the distinctive surface electronic structure, which is a brand new class of promising 2D electrocatalyst for sustainable energy device application. However, achieving such an atomically thin multi-metallene still presents a great challenge. Herein, we present a new synthetic method for an atomic-level palladium-iridium (PdIr) bimetallene with an average thickness of only ∼1.0 nm for achieving superior catalysis in the hydrogen evolution reaction (HER) and the formic acid oxidation reaction (FAOR). The curved PdIr bimetallene presents a top-ranked high electrochemical active area of 127.5 ± 10.8 m^2^ g_Pd+Ir_^−1^ in the reported noble alloy materials, and exhibits a very low overpotential, ultrahigh activity and improved stability for HER and FAOR. DFT calculation reveals that the PdIr bimetallene herein has a unique lattice tangential strain, which can induce surface distortion while concurrently creating a variety of concave-convex featured micro-active regions formed by variously coordinated Pd sites agglomeration. Such a strong strain effect correlates the abnormal on-site active 4d^10^-t_2g_-orbital Coulomb correlation potential and directly elevates orbital-electronegativity exposure within these active regions, resulting in a preeminent barrier-free energetic path for significant enhancement of FAOR and HER catalytic performance.

## Teaser

This work reports the first-time synthesis of palladium-iridium bimetallene with lattice tangential strain, which achieves superior catalysis for hydrogen evolution reaction and formic acid oxidation reaction.

## INTRODUCTION

Electrocatalysis will have a significant role in realizing the widespread use of renewable energy in future [1–3]. However, most catalysts used for renewable energy application still rely largely on the precious metals. To reduce the dependence on and massive depletion of these precious metals, great efforts have been devoted to searching for desirable electrocatalysts with the guidance of both/either increasing exposed active sites *per* mass and/or their intrinsic activity [4–6]. Regulation of the morphology [7–9] and composition [10–12] of catalysts are conventional methods to realize the above goals, with optimization of appropriate atomic number and electron distribution on the surface of these metallic catalysts being key to the achievement of more efficient catalytic performance.

Thin two-dimensional (2D) nanomaterials are a recently identified class of highly efficient catalysts [13–17]. Their merit for electrocatalysis is that most of their metal atoms are exposed on the surface to take part in the catalytic reaction, thus greatly improving the atomic utilization. Meanwhile, the exposure with specific facets on the surface, similar to the typical thin film system [18,19], makes it much easier to identify the active sites on the 2D nanomaterials for electrocatalysis, which can then be modeled by experiments or theoretical calculation. More importantly, thin 2D nanomaterials always exhibit a varied surface intrinsic electronic structure relative to their bulk or nanoparticle counterparts [20], and the overlapping degree of diverse electron orbitals on the surface could be more effectively regulated by atomic surface-modification [21] and/or strain-engineering [22] because of the well-defined thin 2D structures. All these merits of thin 2D nanostructures equip them with good advantages to acquire the desirable surface atomic/electronic structure for electrocatalysis and establish the corresponding surface electronic structure-electrocatalytic property relationship. Very recently, it was observed that atomically thin 2D metals/alloys possess intrinsic strain related to their specific thickness [23] or curvature [24], which greatly modulates the surface electronic structure and energetics of reaction intermediates, which can then promote catalytic activities. However, because of great difficulties in synthesizing such suprathin multi-metallic nanosheets (NSs) at few atomic thickness levels, such a concept is largely constrained in that it has not been well demonstrated or fully discussed.

Herein, we present a new strategy for synthesizing suprathin, curved PdIr alloy NSs with around five atomic layers of thickness, in design of superior catalysts. Because of the structural analogy with graphene, in this study, we refer to the PdIr alloy NSs as ‘PdIr bimetallene’. The PdIr bimetallene shows a very high electrochemically active surface area (ECSA, 127.5 ± 10.8 m^2^ g_Pd+Ir_^−1^) and unique surface electronic structure, enabling superior electrocatalytic performance for both the hydrogen evolution reaction (HER) with reduced overpotential of 34 mV at current density of 10 mA cm^−2^ relative to that of commercial Pt/C (78 mV), and the formic acid oxidation reaction (FAOR) with mass activity of 2.7 A mg_Pd+Ir_^−1^ at 0.5 V *vs* reversible hydrogen electrode (RHE), 4.8 times higher than the commercial Pd/C. DFT calculations confirm that the surface strain effect endows the PdIr bimetallene with exceptional modulation ability of surface electronic structures within the concave-convex featured micro-active regions for higher electroactivity. Thus, excellent HER and FAOR performances originate from the optimized surface binding, in which the Pd-overbinding has been alleviated *via* Ir-5d t_2g_ suppression. Concurrently, the Ir-5d e_g_ activity ensures extraordinarily high OH-dissociation efficiency and stable HCOO*-docking to achieve the prominent FAOR process. Therefore, the realization of bifunctional atomic d-band-tuning engineering has been excavated for optimal electrocatalytic performance of HER and FAOR.

## RESULTS AND DISCUSSION

**Figure 1.**
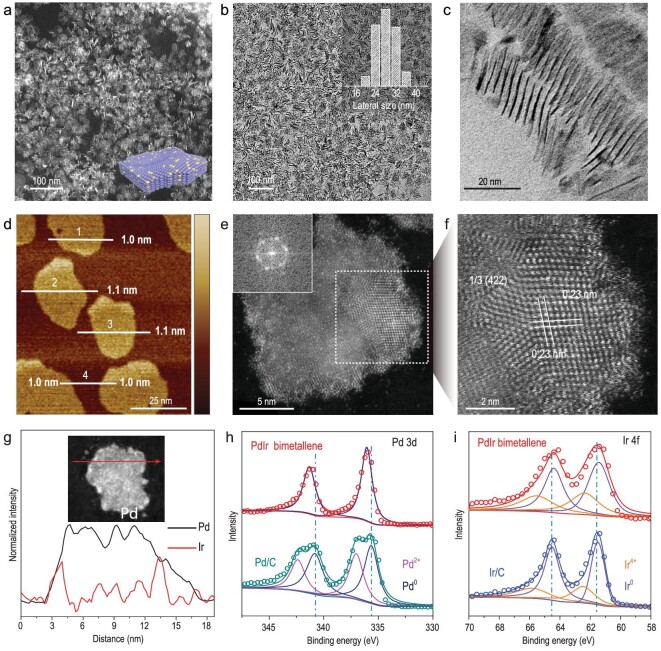
Morphology and phase characterization of PdIr bimetallene. (a) Low-magnification HAADF-STEM image. (b and c) TEM images and the lateral size distribution (inset of Fig. 1b) of PdIr bimetallene. (d) AFM and (e and f) HAADF-STEM images of PdIr bimetallene. (g) EDS linear scan of PdIr bimetallene for Pd and Ir elements. (h and i) XPS spectra of Pd 3d of PdIr bimetallene/C and commercial Pd/C, and Ir 4f of PdIr bimetallene/C and commercial Ir/C.

Typical PdIr bimetallene was synthesized by reduction of palladium acetylacetonate (Pd(acac)_2_) and continuous decomposition of tetrairidium dodecacarbonyl (Ir_4_(CO)_12_) in oleylamine (OAM) at 150°C (details in Methods). Representative low-magnification high-angle annular dark-field scanning transmission microscopy (HAADF-STEM) and transmission electron microscopy (TEM) images show that the obtained products are entirely composed of graphene-like NSs, which are either horizontal or vertical standing. As observed from vertical standing PdIr bimetallene, most have a lateral size of between 20 and 30 nm, with a specially curved structure (Fig. 1a and b). The ultrathin character of the PdIr bimetallene was further confirmed by high-magnification TEM and atomic force microscopy (AFM) measurements. Ultrathin nanostructures are usually very sensitive to electron beams under the common TEM testing environment [25,26]. In our case, pores appeared immediately on the horizontal standing NSs when they were subjected to high-magnification TEM, indicative of suprathin thickness (Fig. S1). In addition, Fig. 1c shows a side view of several groups of NSs with lamellar structures. The thickness of the PdIr bimetallene is measured to be ∼1 nm, about five atomic layers thick, in agreement with high-resolution TEM (HRTEM) (Fig. S2) and AFM results (Fig. 1d). Aberration-corrected HAADF-STEM was performed to present the atomic-scale surface structure of one individual PdIr bimetallene (Fig. 1e). The corresponding fast Fourier transformation (FFT) pattern of the NS (inset of Fig. 1e) shows a 6-fold rotational symmetry structure of the PdIr bimetallene, denoting that the NS has a face-centered cubic (*fcc*) crystal structure stacked with (111) facets in the vertical direction [20]. Distinct lattice spacing of 0.23 nm from the enlarged image of one part of this NS can be assigned to the 1/3 (422) fringe of *fcc* structure (Fig. 1f). To analyze the elemental composition of the PdIr bimetallene, results from STEM energy dispersive X-ray spectroscopy (EDS) line scanning and mapping of single PdIr bimetallene are shown in Figs 1g and S3. The Pd and Ir elements are homogeneously distributed on the NS. The composition ratio of Pd to Ir in PdIr bimetallene is 77.3/22.7, as determined by inductively coupled plasma atomic emission spectroscopy (ICP-AES), consistent with the result of TEM-EDS (78.2/21.8). An X-ray photoelectron spectroscopy (XPS) result (Fig. 1h and i) further reveals that surface Pd and Ir in PdIr bimetallene are mainly in the metallic state, and the peaks of Pd 3d of PdIr bimetallene are clearly shifted to higher binding energy while the peaks of Ir 4f are shifted to lower binding energy, indicating electron transfer from Pd to Ir on the surface of PdIr bimetallene.

**Figure 2.**
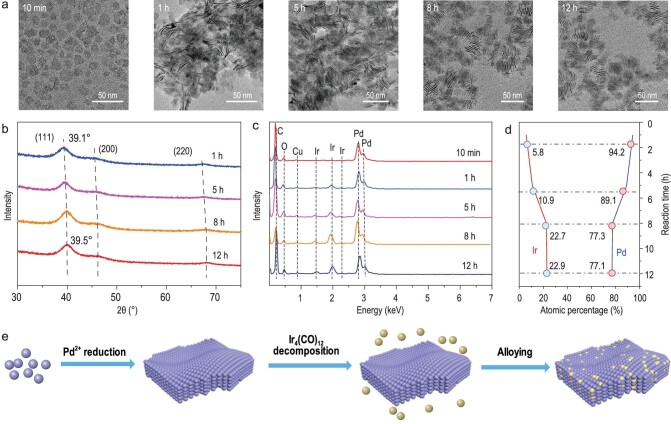
Time-dependent growth process of PdIr bimetallene. (a) TEM images of products at different reaction time of 10 min, 1 h, 5 h, 8 h and 12 h, respectively. (b) XRD patterns of products at different reaction time. (c and d) EDS spectrums and the atomic ratio of Pd and Ir of the products at different reaction time. (e) Schematic illustration of the growth process of PdIr bimetallene.

Time-dependent experiments were carried out to better understand the growth mechanism of PdIr bimetallene. TEM images of the products at different reaction time are displayed in Fig. 2a. The results show that a number of small NSs have already formed at the initial reaction stage (10 min). The lateral size of NSs continued to grow in the next hour, and then their morphology stayed almost unchanged. X-ray diffraction (XRD) and TEM-EDS were also performed to study the phase and elemental composition of these intermediates. The XRD patterns of these products all showed three typical *fcc* peaks and the peaks continuously shifted to higher angles as the reaction time proceeded from 1 to 8 h, indicating that more Ir atoms were alloyed with Pd metallene during the post-reaction stage (Fig. 2b). The alloying process could also be traced from the atomic ratio of Pd and Ir, as calculated from TEM-EDS (Fig. 2c and d). At the first 10 min reaction, the obtained small NSs were basically composed of Pd. After that, the Ir(0) atoms, decomposed from Ir_4_(CO)_12_, began to embed into the Pd metallene. The achieved highest atomic percentage of Ir element in PdIr bimetallene is almost 23% after 8 h, and did not further increase when extending reaction time to 12 h. When we changed the dosage of Ir_4_(CO)_12_ in the precursor, the atomic ratio of Pd/Ir in bimetallene could be tuned (Fig. S4). From ultraviolet-visible (UV-VIS) spectrums of the intermediate products, their absorption peaks show a negative shift over reaction time, attributed to change of surface electronic structure on the PdIr bimetallene during the alloying process (Fig. S5). Furthermore, we found that usage of Ir_4_(CO)_12_ was necessary for formation of PdIr bimetallene. When substituting the Ir_4_(CO)_12_ with other iridium precursors and keeping other conditions consistent, a mixture of only irregular Pd NSs and tetrahedral Pd nanostructures was obtained (Fig. S6). Reduction of dissolved Ir(^3+^) to Ir(^0^) atoms in OAM solvent always required a high temperature (>200°C) [8,27], whereas Ir was detected in the products with the use of Ir_4_(CO)_12_ even when the temperature was lowered to 100°C (Fig. S7). In addition, the CO decomposed from the W(CO)_6_ and Ir_4_(CO)_12_ in our method, served as a surface capping-agent, confining the growth of Pd (111) facets and resulting in the 2D structure [28–30]. Otherwise, PdIr nanoparticles (PdIr NP) were generated without adding W(CO)_6_ (Fig. S8). We infer that the synthetic process of PdIr bimetallene relied upon first reduction of Pd(acac)_2_ to form Pd metallene, then decomposition of Ir_4_(CO)_12_ and finally diffusion of Ir(0) atoms to the Pd NSs (Fig. 2e), similar to recently reported PdMo bimetallene [24]. However, the Ir element is much more stable than the Mo element and can exist on the outer surface of PdIr bimetallene, perhaps participating in the catalytic process as synergistic active sites with Pd. Besides, based on the XRD measurements (Fig. S9), we noted that the peak indexed to the (111) plane of PdIr bimetallene was located at 39.5°, smaller than those of the synthesized PdIr NP with identical atomic Pd/Ir ratio (40.2°), standard *fcc* Pd (40.11°, JCPDS 46-1043) and Ir crystal (40.66°, JCPDS 06-0598). Extended X-ray adsorption fine structure (EXAFS) analysis (Fig. S10 and Table S1) also revealed that the length of the Pd-Pd(Ir) bond in PdIr metallene is about 0.277 nm, larger than those in PdIr (0.274 nm) and Pd foil (0.274 nm). The strain of PdIr bimetallene is therefore calculated to be 1.1%.

To further confirm the ultrathin structure of PdIr bimetallene, the ECSA of the carbon supported PdIr bimetallene (PdIr bimetallene/C, Fig. S11) was firstly tested by cyclic voltammetry (CV) in 0.1 M HClO_4_ by integrating the charge of the H_upd_ desorption peak (Fig. S12). PdIr bimetallene/C showed an ECSA of 130.3 ± 9.2 m^2^ g_Pd+Ir_^−1^, much larger than those of carbon supported PdIr NP (PdIr NP/C, 38.9 ± 5.0 m^2^ g_Pd+Ir_^−1^), commercial Pd/C (38.6 ± 2.8 m^2^ g_Pd_^−1^) and Pt/C (70.1 ± 4.3 m^2^ g_Pt_^−1^). Pd can easily absorb hydrogen into its inner lattice, resulting in inaccuracies to the calculated ECSA. Thus, we applied CO stripping methods to calculate the ECSA of PdIr bimetallene/C, PdIr NP/C and commercial Pd/C (Fig. S13). The calculated ECSA results are summarized in Table S2, and the value of our synthesized PdIr bimetallene (127.5 ± 10.8 m^2^ g_Pd+Ir_^−1^) still ranks the highest among recently reported Pd-based catalysts (Table S3).

**Figure 3.**
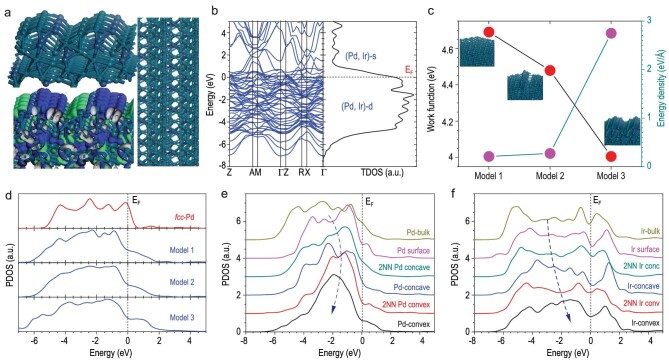
Theoretical calculation of the electron structure of the PdIr bimetallene. (a) The strained (111) surface lattice of the as-built Pd_8_Ir_2_ structural model (model 3) with charge density distributions. (b) Band structure and total density of states (TDOSs) of the Pd_8_Ir_2_ model. (c) Surface work functions and energy area density dependences are shown for three given surface models. (d) PDOSs of overall d-bands for *fcc*-Pd, model 1, 2 and 3 for illustrating strain modulation. (e) PDOSs of 4d bands of variously coordinated Pd sites exposing within concave-convex region. (f) PDOSs of 5d bands of variously coordinated Ir sites nearby those modulated Pd sites.

Inspired by the atomically thin thickness and intrinsic strain resulting from the curve structure of PdIr bimetallene, we employed DFT calculations to project its underlying unique surface electronic structure. According to the above structure characterization of PdIr bimetallene, the surface (111) of Pd_8_Ir_2_ structural model has been constructed with five-layer thickness. In creating the large dislocations and the generated strain applied parallel [111]-direction, there have been obvious (Pd-Ir)-reconstructed concave-convex featured distortions formed on the Pd_8_Ir_2_ (111) surface with strip ripples (Fig. 3a). Local charge densities from electronic states near the Fermi level (E_F_) reflect that the concave-convex regions are the electro-active areas. The distortion character reflects a continuous and smooth variation trend in local features of the density of states (DOSs), showing clear tail states across the E_F_. This confirms the absence of long-range order in the lattice (Fig. 3b). With strain scale increased, the energy area density increases while the surface work function exhibits the inverse behavior, denoting enhanced electronic activity on the surface. This trend indicates that the surface on-site orbital Coulomb potential has been modulated as the electronic-activation barrier was lowered, showing a direct correlation with surface strain effect (Fig. 3c). Further analysis on the projected partial density of states (PDOSs) interprets that the overall d-band has been clearly modulated by the surface strain, shifting the lower position to alleviate the overbinding effect (Fig. 3d). We see that the surface strain effect evidently tunes the Pd-4d electronic characters from concave to convex to increase the selectivity. The 4d band center has downshifted from E_V_-1.7 eV (concave) to E_V_-2.6 eV (convex), referred to the strain-free PdIr surface (E_V_-1.5 eV). Even the second nearest neighboring (2NN) Pd sites exhibited an obvious response to the strain-induced 4d variations (Fig. 3e). More contrast variation trend has been illustrated in Fig. S14. We further studied the role of Ir among different regions. The variation trend of the 5d-band implies an opposite trend to that of the related nearby Pd site. It indicates that the Ir-5d orbital not only disturbs the inter-d-orbital Coulomb potential distribution for generating the strain, but also acts as an electron relay center to associate site-to-site electron transfer of Pd site (Fig. 3f), which is a deep interpretation from the XPS results. And the surface strain causes the Ir-5d_eg_-component to stay empty above E_F_ for coordinating H_2_O or HCOOH (Fig. S15) in facilitating dissociation of H_2_O or HCOOH.

**Figure 4.**
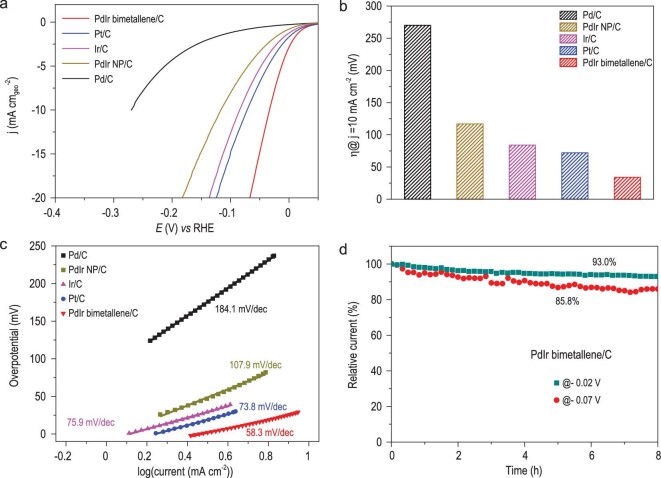
HER activity and durability of PdIr bimetallene in 0.1 M KOH. (a) HER polarization curves of PdIr bimetallene/C, PdIr NP/C, Ir/C, Pt/C and Pd/C at a scan rate of 5 mV s^−1^ with 95% iR-compensation. (b and c) Overpotentials at 10 mA cm^−2^ (b) and Tafel slopes (c) of different catalysts. (d) Chronoamperometric curves of the PdIr bimetallene/C catalysts at an applied potential of −0.02 and −0.07 V *vs* RHE.

As a substitute for Pt in the hydrogen evolution reaction (HER), Pd metal has higher reserves on Earth and exhibits suitable H-bonding energy near the peak of the volcano plot, although its performance is still not comparable with that of Pt [31]. To prove the advantage of our synthesized PdIr bimetallene for electrocatalysis, we firstly conducted HER tests of PdIr bimetallene with different Pd/Ir atomic ratios in alkaline media (Figs S16 and S17). The result shows that Ir alloying can indeed promote the HER activity of pure Pd metallene. An atomic ratio of Pd/Ir at 4 : 1 in the PdIr bimetallene can lead to the best HER activity. Surprisingly, the optimized PdIr bimetallene/C also shows the highest onset potential among the other five catalysts, with the order of PdIr bimetallene/C > Pt/C > Ir/C > Pd/C > PdIr NP/C > Pd/C (Fig. 4a). To achieve the current density of 10 mA cm^−2^, the PdIr bimetallene/C required only an overpotential of 34 mV, much smaller than that of Pt/C (72 mV), Ir/C (84 mV), PdIr NP/C (117 mV) and Pd/C (270 mV) (Fig. 4b). In electrocatalysis, the smaller Tafel slopes of the catalysts reflect the higher kinetic efficiency. The PdIr bimetallene/C shows a Tafel slope of 58.3 mV/dec, much lower than that of PdIr NP/C catalyst (107.9 mV/dec) and even lower than that of Pt/C (73.8 mV/dec), indicating accelerated HER dynamics on the PdIr bimetallene (Fig. 4c). PdIr bimetallene exhibits the highest specific activity and mass activity among those catalysts (Figs S18 and S19), in particular delivering a mass activity of 2.67 mA μg_Pd_^−1^ (2.06 mA μg_Pd+Ir_^−1^), 2.8, 4.7 times and 33.3 times higher than those of commercial Pt/C (0.92 mA μg_Pt_^−1^), PdIr NP (0.56 mA μg_Pd+Ir_^−1^) and Pd/C (0.08 mA μg_Pd_^−1^), presenting the best level of the HER catalysis in alkaline solution yet reported (Table S4). As is known, the HER involves two sequential steps in alkaline media: dissociation of water to get protons and recombination of protons to release H_2_ [32]. The first step is the rate-determining step with sluggish kinetics, leading to poorer HER performance in alkaline media than in acid [32–34]. As a consequence, the origin of the superb HER activity of PdIr bimetallene could be attributed to the enhanced water-dissociation ability of the protrusion Pd-Ir reconstructed site on the PdIr bimetallene. In accordance with the theoretical calculations, the surface Ir sites could facilitate dissociation of the OH group from the H_2_O to produce enough protons (Fig. S14), and then the protons are adsorbed and combined on the Pd sites, further accounting for the enhancement of the HER activity of PdIr bimetallene over Pd metallene.

The chronoamperometry technique was employed to examine the electrocatalytic stability of PdIr bimetallene. Figure 4d shows time-dependent current density retention curves of the PdIr bimetallene under static overpotential of 20 and 70 mV, respectively. After an 8 h durability test, PdIr bimetallene/C maintained 93.0% and 85.8% percentages of its original activity at 20 and 70 mV *vs* RHE, much higher than similarly calculated for commercial Pt/C (55.4% and 38.1%, Fig. S20). The Pt nanoparticle had undergone severe aggregation, whereas PdIr bimetallene was well preserved after stability testing (Fig. S21). The stability enhancement on PdIr bimetallene can be attributed to its stronger binding with carbon support than Pt nanoparticle, realized by more direct contact through the NSs structure. Meanwhile, the surface of PdIr metallene contains more atoms with low coordination, which are more stable in catalytic reaction. To further prove the stability of PdIr bimetallene over a longer time, instead of using GC electrode, we retested the HER of the PdIr bimetallene with mass loading of 0.2 mg_Pd+Ir_ cm^−2^ using carbon paper (CP) as electrode substrate. The chronopotentiometry of PdIr bimetallene at 10 mA cm^−2^ (Fig. S22) showed very little decay after 75 h testing, revealing the super stability of PdIr bimetallene for HER.

**Figure 5.**
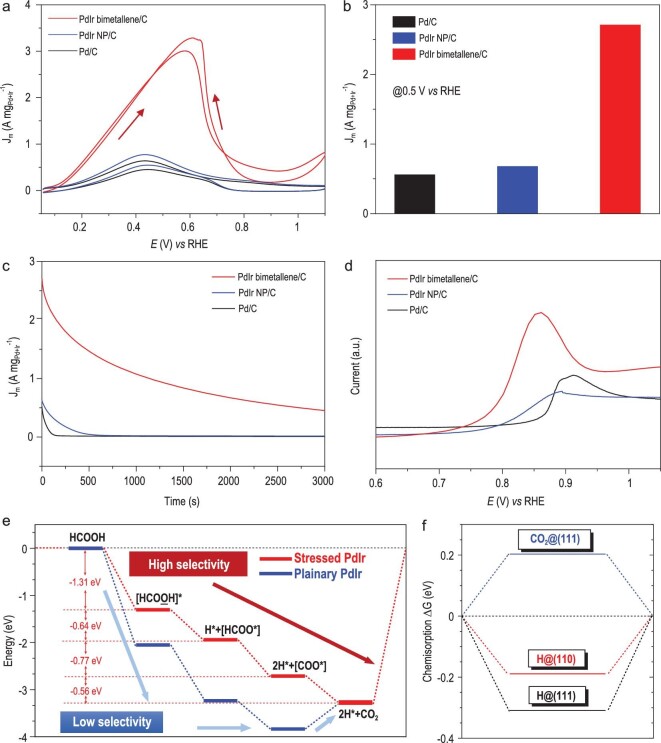
FAOR performance and enhancement mechanism of PdIr bimetallene. (a) CVs of PdIr bimetallene/C, PdIr NP/C and Pd/C for FAOR in 0.1 M HClO_4_ containing 0.5 M HCOOH at the scan rate of 50 mV s^−1^. (b) Mass activities of different catalysts at the potential of 0.5 V *vs* RHE. (c) Chronoamperometric curves of the PdIr bimetallene/C, PdIr NP/C and Pd/C catalysts at 0.5 V *vs* RHE. (d) CO stripping tests of PdIr bimetallene/C, PdIr NP/C and Pd/C catalysts conducted in 0.1 M HClO_4_ at the scan rate of 50 mV s^−1^. (e) Free energy pathway (ΔG) for FAOR under acidic conditions. (f) Chemisorption of H and CO_2_ on the surface (111).

As further proof of the superiority in electrocatalysis of large atomic exposure area with unique electronic structure in our synthesized PdIr bimetallene, we examined the electrocatalytic performance of PdIr bimetallene/C, Pd metallene/C, PdIr NP/C and commercial Pd/C in the formic acid oxidation reaction (FAOR). The onset potential of PdIr bimetallenes all shift negatively compared with that of Pd metallene, revealing that Ir alloying on Pd metallene can accelerate the FAOR (Fig. S23). From the typical FAOR curves of optimal PdIr bimetallene/C, PdIr NP/C and commercial Pd/C (Figs 5a and S24), the PdIr bimetallene/C exhibits the lowest onset potential and highest specific activity among all the investigated catalysts, indicating its excellent intrinsic activity. Meanwhile, benefiting from the high atomic utilization, the mass activity of PdIr bimetallene/C could reach up to 2.70 A mg_Pd+Ir_^−1^ at a potential of 0.5 V *vs* RHE, 3.97 and 4.82 times higher than those of PdIr NP/C (0.68 A mg_Pd+Ir_^−1^) and Pd/C (0.56 A mg_Pd_^−1^) (Fig. 5b). Compared with other reported Pd-based FAOR catalysts, its mass activity at peak is about 3.75 A mg_Pd_^−1^, presenting the top level (Table S5).

The electrocatalytic stability of these three catalysts was further measured by chronoamperometric measurements at a constant potential of 0.5 V *vs* RHE. In Fig. 5c, the drop speeds follow with the order of PdIr bimetallene/C < PdIr NP/C < Pd/C, while the PdIr bimetallene/C is the most stable showing mass activity remaining at 0.45 A mg_Pd+Ir_^−1^ after 3000 s. We examined the FAOR activity again in the refreshed electrolyte after the durability test (Figs S25 and S26). We found that the mass activity of PdIr bimetallene/C remained at almost 78% at a potential of 0.5 V *vs* RHE, much higher than those of Pd metallene/C (42%), PdIr NP/C (28%) and commercial Pd/C (15%). The TEM images also show that the structure of the PdIr bimetallene is basically unchanged after the FAOR (Fig. S27).

Pathways of the FAOR on the catalysts are usually of two kinds: dehydrogenation or dehydration [35]. For the Pd-based catalysts, dehydrogenation is often the main pathway, but recent works indicate that the dehydration pathway also occurs on Pd [36]. In the latter pathway, the reaction intermediate CO adsorbs and then assembles on the surface of Pd, hindering further oxidation of the formic acid molecule, leading to fast degradation of catalytic performance under long-term FAOR conditions [37,38]. To understand the reasons for stability improvement of PdIr metallene, the CO tolerance of these catalysts was examined using the CO stripping test (Fig. 5d). It can be observed clearly that the CO oxidation peaks on PdIr NP/C show a slight negative shift compared with that on Pd/C, revealing that CO oxidation is easier on Pd when it is alloyed with Ir. Surprisingly, the CO anti-poisoning ability can be further promoted on the PdIr bimetallene, revealed by the extra negative shift of CO oxidation peaks compared with PdIr NP/C. Usually Ir metal does not possess the ability to promote electrocatalytic oxidation of formic acid while Pd metal is the active site [39]. In the case of PdIr bimetallene, with its optimal surface electronic structure, the Pd-Ir protrusions can provide extraordinarily high performance in OH dissociation and efficient docking of the leaving group (HCOO*). The surface Ir-5d band actually provides substantial overlapping with the O-2p band, and a few overlaps with the C-2p band given from the orbital components from the HCOOH molecule (Fig. S14). This indicates that Ir sites on the top protrusion surface area can stably locate the O-part from the HCOOH for straightforward acidic FAOR with a large energetic gain, instead of adsorption of C site towards a catalyst poisoning reaction. Meanwhile, the Ir-O bond can further prevent local oxidation of the Pd (II) site based on promoted site-to-site electron transfer, which is significant as an electrogeneration center for enhancing HCOOH electro-oxidation.

To shed further light on the catalytic mechanism of PdIr bimetallene, the energetic pathway for FAOR was studied under acidic conditions, showing that the overall reaction heat is in a continuous downtrend with spontaneous character of ΔG < 0. With the reference level infinitely far away, the itinerant HCOOH molecule anchoring on the Pd_8_Ir_2_ (111) surface gains an energy of −1.31 eV. This is potentially the rate-determining step as it possesses the largest energy gain overall for the FAOR process. Further strong surface adsorption reflects the OH bond dissociation with −0.64 eV decreased. Meanwhile, we found that the surface Pd-Ir protrusions enhanced further splitting of the OH that produces the step [COOH→H*+COO*], gaining extra energy of −0.77 eV with the existence of adsorbing *H. In the final step when the local stably adsorbed 2H* still remained, the adsorbed COO* turned to form CO_2_ gas molecules to be desorbed from the surface. In contrast to the strain-free PdIr surface, the FAOR process is more selective and energetically preferred to proceed with −0.56 eV lowered (Fig. 5e). We compared the chemisorption energies of H and CO_2_, respectively. It was found that H at the (111) surface reflects a negative chemisorption ΔG, denoting a potential rather stable initial adsorption towards high H-coverage. However, the CO_2_ chemisorption energy shows positive chemisorption above the thermo-neutral line (ΔG = 0), which implies efficient desorption of CO_2_. This energetic comparison can natively show suppression of further poisoning of the catalyst and secondary CO_2_ adsorption for oxidizing the active Pd site (Fig. 5f). Therefore, this trend guarantees an efficient FAOR and further fast rate for the HER under small overpotential. From the local structure (Fig. S27), the stable adsorbed H is bonding with the Pd site, and the HCOO group is also stably adsorbing on the Ir site at the top surface of the protrusion area. The bond-cleavage of the HO process from HCOOH occurs between the Pd and Ir sites. The two closely adsorbed *H on the Pd sites actually contribute to the potentially efficient H_2_ generation. With assistance from the Ir site, the leaving group (HCOO*) can be stably located on the surface of Pd_8_Ir_2_ (111).

## CONCLUSION

In summary, we report a new class of PdIr bimetallenes as an extraordinary electrocatalyst for both HER and FAOR. Super-high atom exposure and strain-induced unique electron structure on the surface contribute to the exceptional mass activity for HER in alkaline media, 33.3 times higher than that of Pd/C, representing the best reported performance of Pt-free HER catalyst yet. Furthermore, the PdIr bimetallene shows superior activity and stability over home-made PdIr NP and commercial Pd/C towards FAOR in acid media. DFT calculations unravel formation of concave-convex featured electro-active regions by the strong surface strain effect, which directly correlate with the unique on-site 4d^10^-t_2g_-orbital Coulomb potential and propel higher orbital-electronegativity within these active regions. Such modulation has optimized the electronic environment for energetically favorable evolution of actualizing the universally excellent HER and FAOR performance. Beyond the conventional modulation of nanocatalysts, we believe the proposed strategy will innovate new inspirations for pursuing suprathin 2D multi-metallic nanostructures with the desired surface structure and composition to extend potential applications in various fields.

## METHODS

### Synthesis of PdIr bimetallene

In a typical preparation of PdIr bimetallene, 10 mg Pd(acac)_2_, 2.5 mg Ir_4_(CO)_12_, 30 mg W(CO)_6_, 25 mg NH_4_Br and 5 mL OAM were added to a 15 mL pressure bottle, and ultrasonicated to obtain a homogeneous solution. After the pressure bottle was sealed, the mixture was heated to 150°C and maintained at 150°C for 8 h in an oil bath. The cooled product was washed three times with a cyclohexane/ethanol mixture (v : v, 5 : 1) to wash off the redundant OAM. Note, the airtightness of the reaction vessel is essential to prevent volatilization of Ir_4_(CO)_12_ in reaction, otherwise the atomic radio of Pd/Ir element in end products will be much larger than that of added Pd/Ir precursor.

### Electrochemical tests

The carbon supported catalysts were dispersed in a mixture containing water, isopropanol and 5 wt% Nafion (volume ratio: 0.75 : 0.24 : 0.01) to form homogeneous ink by sonication for 60 min in an ice bath. The concentration of precious metal was controlled to be 0.2 mg_metal_ mL^−1^ based on the ICP-AES measurement. Electrochemical tests were performed using a CHI660E electrochemical workstation (Chenhua, Shanghai) with a three-electrode system. The catalyst modified rotating disk glass carbon (GC) electrode (Pine Ins) was used as the working electrode, an Ag/AgCl (in acid) or Hg/Hg_2_Cl_2_ (in base) electrode was used as the reference electrode and a Pt wire (for FAOR) or graphite rod electrode (for HER) was used as the counter electrode. All measurements were conducted at room temperture. HER tests were carried out in N_2_-saturated 0.1 M KOH, and the linear sweep voltammetry was recorded at a scan rate of 5 mV s^−1^ with a rotation rate of 2000 rmp. The mass loading was controlled to be ∼2 μg_metal_ on the electrode, determined by ICP-AES. The measured resistance of 0.1 M KOH was around 43.6 Ω and 95% iR drop compensation was conducted unless otherwise mentioned. Before the FAOR tests, a surface cleaning step of the catalysts was applied by CV in N_2_-saturated 0.1 M HClO_4_ at high sweep rate of 500 mV s^−1^ at a potential of 0.05–1.0 V *vs* RHE. Then, the FAOR tests were performed in N_2_-saturated 0.1 M HClO_4_ + 0.5 M HCOOH. Cyclic voltammetry was applied to evaluate the performance of catalysts at a rate of 50 mV s^−1^. The chronoamperometry technique was used to investagate the stability of catalysts. The ECSA was determined by integrating the charge of underpotentially deposited H and stripping of CO. All electrochemical data were tested for at least three times. For the CO stripping tests, the experiments were carried out in 0.1 M HClO_4_. Before the test, the solution was purged with Ar for 20 min to remove the O_2_ in the solution, and then bubbled with CO gas (99.9%) for 10 min at 0.1 V *vs* RHE. The residual CO in the solution was excluded by Ar for another 20 min.

## Supplementary Material

nwab019_Supplemental_File
