# Supplementary material for: A highly efficient atomically thin curved PdIr bimetallene electrocatalyst
Source: Natl Sci Rev. 2021 Feb 2;8(9):nwab019. doi: 10.1093/nsr/nwab019 (PMC8433090; doi:10.1093/nsr/nwab019)
Supplement: nwab019_Supplemental_File [file nwab019_supplemental_file.doc]

**Supporting Information for**

**A Highly Efficient Atomically-thin Curved PdIr Bimetallene Electrocatalyst**

Fan Lv^#^,^1,2^ Bolong Huang^#^,^3^ Jianrui Feng,^1^ Weiyu Zhang,^1^ Kai Wang,1 Na Li,^4,5^ Jinhui Zhou,^1^ Peng Zhou^1^, Wenxiu Yang,^1^ Yaping Du,^6^ Dong Su,^5^ Shaojun Guo^*1,2^

^1^College of Engineering, Peking University, Beijing 100871, P. R. China.

^2^ School of Materials Science and Engineering, Peking University, Beijing 100871, P. R. China.

^3^Department of Applied Biology and Chemical Technology, Hong Kong Polytechnic University, Hung Hom, Kowloon, Hong Kong SAR.

^4^Frontier Institute of Science and Technology jointly with College of Science, Xi’an Jiaotong University, Xi'an, Shaanxi Province, 710054, China.

^5^Center for Functional Nanomaterials Brookhaven National Laboratory Upton, New York 11973, United States

^6^School of Materials Science and Engineering & National Institute for Advanced Materials, Nankai University, Tianjin 300350, China.

^#^These authors contributed equally to this work.

*Correspondence: [guosj@pku.edu.cn](mailto:guosj@pku.edu.cn)

**Methods**

**Chemicals.** Palladium acetylacetonate [Pd(acac)_2_, 99%], tetrairidium dodecacarbonyl [Ir_4_(CO)_12_, 98%], hexacarbonyltungsten [W(CO)_6_, 97%], oleylamine (OAM, 68-70%) and commercial Pd/C (10 wt% of 8 nm Pd nanoparticles on activated carbon) were all purchased from Sigma-Aldrich. Commercial Pt/C (20 wt%, 3-nm Pt nanoparticles on carbon black) was purchased from JM Corporation. Commercial Ir/C (20 wt%, 2-4 nm Ir nanoparticles on carbon black) was purchased from Premetek Company. Ammonium bromide (NH_4_Br, 99.0%), cyclohexane(C_6_H_12_, 99.5%), ethanol(C_2_H_6_O, 99.5%), isopropanol (C_3_H_8_O, 99.5%) and formic acid (CH_2_O_2_, 98%) were purchased from Sinopharm Chemical Reagent Co. Ltd. L-ascorbic acid (AA, 98%) was purchased from J&K Scientific Ltd. Nafion solution was obtained from Alfa Aesar. Potassium hydroxide (KOH, 95%) and perchloric acid (HClO_4_, 70-72%) were purchased from Aladdin Reagent Co. Ltd. All the chemicals were used without further purification.

**Synthesis of PdIr NPs.** 10 mg of Pd(acac)_2_, 2 mg of Ir_4_(CO)_12_, 25 mg of NH_4_Br, 15 mg of AA and 5 mL of OAM were added into a 20 mL vial and ultrasonicated for around 1 h to get the homogeneous solution. After the vial was sealed, the mixture was heated to 150 ^o^C within 30 min and maintained at this temperature for 3 h in an oil bath. The cooled product was washed with a cyclohexane/ethanol mixture (v:v, 5:1) to wash off the redundant OAM, and collected by centrifugation at 9500 rpm. The atomic radio of Pd and Ir of synthesized PdIr NPs was all control to be basically identical to 7:2.

**Synthesis of PdIr bimetallene/C and PdIr NPs/C catalysts.** Firstly, 5 mg of PdIr bimetallene or PdIr NPs was dispersed in 60 mL of cyclohexane, and then mixed with 20 mg of Ketjen carbon suspended in 10 mL of ethanol, followed by sonicating for 60 min. After being contrifugated and washed for three times with ethanol, the collected products were dried at 80 ^o^C overnight.

**Materials Characterization.**

Transmission electron microscopy (TEM) was conducted on an FEI Tacnai T20 transmission electron microscope at an acceleration voltage of 120 kV. High-resolution TEM (HRTEM) and TEM energy dispersive X-ray spectroscopy (TEM-EDS) were conducted on JEM-2100F transmission electron microscope at an acceleration voltage of 200 kV. High angle annular dark field scanning TEM (HAADF-STEM) and elemental mapping/line scanning were done on an aberration corrected Hitachi HD2700C at an acceleration voltage of 200 kV. X-ray diffractometer (XRD) was collected on a PANalytical-XRD using a Cu K radiation (λ = 0.15406 nm) at 40 kV voltage and 30 mA current. Atomic force microscopy (AFM) was measured by a Multimode Nanoscope Ⅲa SPA (Veeco Instruments, Bruker). X-ray photoelectron spectroscopy (XPS) tests were done with Kratos AXIS Supra/Ultra spectrometer. The composition and concentration of catalyst were determined by the inductively coupled plasma atomic emission spectroscopy (710-ES, Varian, ICP-AES). The sample for ICP-AES were pre-treated in microwave digestion & extraction system (MARS Xpress). The UV-Vis-near IR absorption spectra of the PdIr bimetallene (dispersed in cyclohexane) were collected by UH4250 spectrophotometer (HITACHI). The catalysts after the durability tests were scratched off the glassy carbon electrode by sonication in ethanol, and then collected for further TEM characterization.

**EXAFS experiment and data processing.**

EXAFS measurements at the Pd K edge in both transmission (for Pd foil) and fluorescence (for samples) mode were performed at beamline BL14W1 at the Shanghai Synchrotron Radiation Facility. The electron beam energy was 3.5 GeV, and the stored current was 260 mA (top-up). A 38-pole wiggler with a maximum magnetic field of 1.2 T inserted in the straight section of the storage ring was used. EXAFS data were collected using a fixed-exit double-crystal Si(311) monochromator. A Lytle detector was used to collect the fluorescence signal, and the energy was calibrated using the Pd foil. The raw data analysis was performed using the IFEFFIT software package according to the standard data analysis procedures. The Fourier transformation of the k^2^-weighted EXAFS oscillations, k^2^χ(k), from k space to R space was performed over a range of 3-11.5 Å^-1^ to obtain a radial distribution function. Data fitting was performed using the Artemis program in IFEFFIT. The passive electron reduction factor S_0_^2^ was 0.78 for Pd foil and was set as 0.85 for other samples.

**Calculation Setup.**

The simple rotationally invariant (Anisimov type) DFT+U functional [1] that embedded in the CASTEP code [2] has been used. The Hubbard U parameter self-consistently determined for the Pd-4d and Ir-5d orbitals by our new linear response method [3]. This method has been already successfully reflecting the on-site orbital Coulomb potential for the transition metal and rare earth elements within DFT+U [4]. The geometry optimization has been performed with the algorithm of Broyden-Fletcher-Goldfarb-Shannon (BFGS). The PBE+U functional was chosen with a kinetic cutoff energy of 750 eV, with the valence electron states expressed in a plane-wave basis set. The ensemble DFT (EDFT) method of Marzari et al [5]. was used for improving the convergence on the Pd and Ir transition metal compounds. The Pd-Ir (111) surface model was built with five-layer thick and atomic size of 108 atoms (i.e. Pd_84_Ir_24_ in 2×2×1 supercell) containing vacuum thickness of 15 Å. Only the lattices in the top two layers are allowed to be freely relaxed. The Monkhost-Pack reciprocal space integration was performed using the mesh of 2×2×1 [6] with Gamma-center-off, which was self-consistently selected for total energy minimization. With these special k-points, the total energy is converged to less than 5.0x10^-7^ eV per atom. The Hellmann-Feynman forces on the atom were converged to less than 0.001 eV/Å. The Pd and Ir norm-conserving pseudopotentials were generated by OPIUM code based on the Kleinman-Bylander projector form [7], together with the non-linear partial core correction [8] and a scalar relativistic averaging scheme [9]. These treatments were used for averaging the spin-orbital coupling effect. We chose the (4d, 5s, 5p) and (5d, 6s, 6p) states as the valence states of Pd and Ir atoms, respectively. The RRKJ method was chosen for the optimization of the pseudopotentials [46]. The Hubbard U parameter on the Rh-4d orbitals was self-consistently to be U_d_=6.13 eV, and U_d_=2.10 eV for Ir-5d.

1. Anisimov VI, Aryasetiawan F and Lichtenstein AI. First-principles calculations of the electronic structure and spectra of strongly correlated systems: the LDA+U method. *J Phy. Condens Matter* 1997; **9**: 767.

2. Clark SJ, Segall MD and Pickard CJ *et al.* First principles methods using CASTEP. *Z Kristallogr* 2005; **220**: 567-70.

3. Huang BL. 4f fine‐-structure levels as the dominant error in the electronic structures of binary lanthanide oxides. *J Comput Chem* 2016; **37**: 825-35.

4. Marzari N, Vanderbilt D and Payne MC. Ensemble density-functional theory for ab initio molecular dynamics of metals and finite-temperature insulators. *Phys Rev Lett* 1997; **79**: 1337-40.

5. Probert MIJ and Payne MC. Improving the convergence of defect calculations in supercells: An ab initio study of the neutral silicon vacancy. *Phys Rev B* 2003; **67**: 075204-11.

6. Kleinman L and Bylander DM. Efficacious form for model pseudopotentials. *Phys Rev Lett* 1982; **48**: 1425-8.

7. Louie SG, Froyen S and Cohen ML. Nonlinear ionic pseudopotentials in spin-density-functional calculations. *Phys Rev B* 1982; **26**:1738-42.

8. Grinberg I, Ramer NJ and Rappe, AM. Transferable relativistic dirac-slater pseudopotentials. *Phys Rev B* 2000; **62**: 2311-4.

9. Rappe AM, Rabe KM and Kaxiras E *et al.* Optimized pseudopotentials. *Phys Rev B* 1990; **41**: 1227-30.

**Supplementary Figures**

**Figure S1.** TEM images of PdIr bimetallene after 1 s (a) and 10 s (b) of electron beam radiation.

**Figure S2.** HRTEM image of PdIr bimetallene supported on Ketjen carbon.

**Figure S3.** (a) EDS mapping and spectra of PdIr bimetallenes.


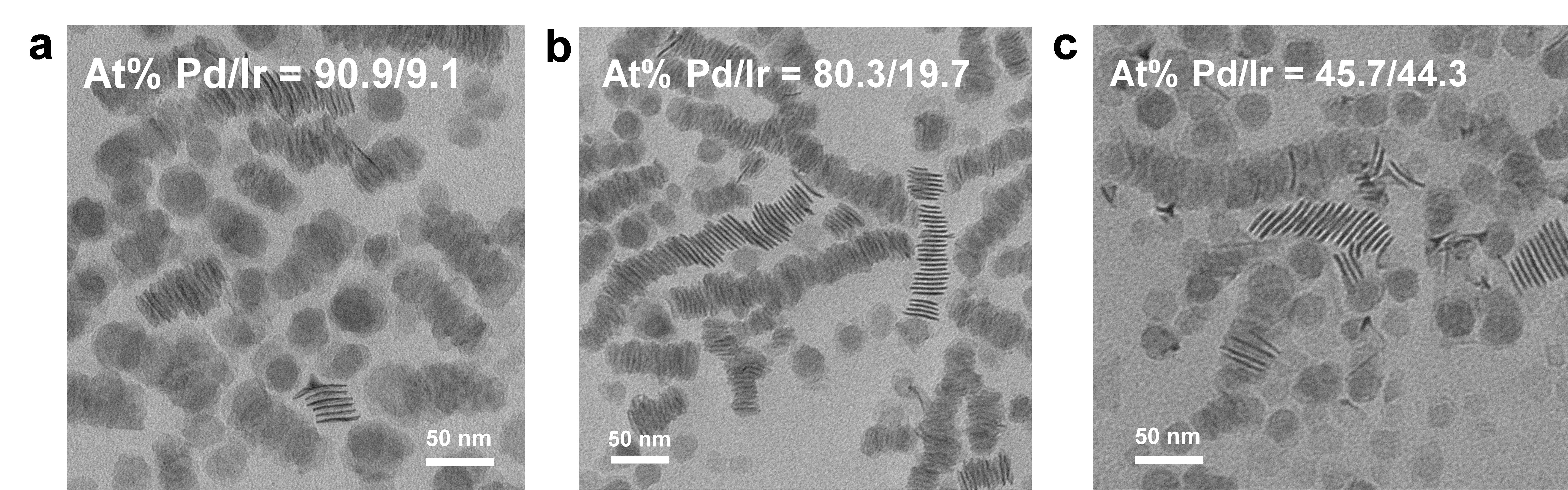


**Figure S4.** TEM images of PdIr bimetallene with different Pd/Ir atomic radio (determined by ICP-AES).

**Figure S5.** (a) UV-VIS spectroscopy of the intermediate product. (b) Optical photograph of PdIr bimetallene dispersed in cyclohexane.

**Figure S6.** (a, c) TEM images and (b, d) EDS spectroscopy of products using Ir(acac)_3_ (a, b) and IrCl_3_·xH_2_O (c, d) as the iridium precursors.

**Figure S7.** (a) TEM image and (b) EDS spectrum of products synthesized at 100 ^o^C.

**Figure S8.** TEM images of products synthesized without adding W(CO)_6_.

**Figure S9**. The XRD patterns and calculated lattice parameters of PdIr bimetallene and Pd Ir NP.

**Figure S10**. The *k*^2^-weighted χ(k)-function of the EXAFS spectra for PdIr bimetallene (red), PdIr NP (blue) and Pd foil (black) without phase-correction.

**Figure S11.** TEM images of carbon supported (a, b) PdIr bimetallene and (c, d) PdIr NPs at different magnification.

**Figure S12.** CVs of (a) PdIr bimetallene/C, (b) PdIr NPs/C, (c) commercial Pd/C and (d) commercial Pt/C in 0.1 M HClO_4_ at the scan rate of 50 mV/s.

**Figure S13.** CO stripping voltammograms of (a) PdIr bimetallene/C, (b) PdIr NPs/C and (c) commercial Pd/C in 0.1 M HClO_4_ at the scan rate of 50 mV/s.

**Figure S14.** The PDOSs of the Pd-4d bands from the surface protrusion, surface, and deep in the bulk regions, respectively.

**Figure S15.** The PDOSs of the Ir-5d, O-2p and C-2p bands when H_2_O (a) and HCOOH (b) molecule adsorbs near the surface protrusion area.


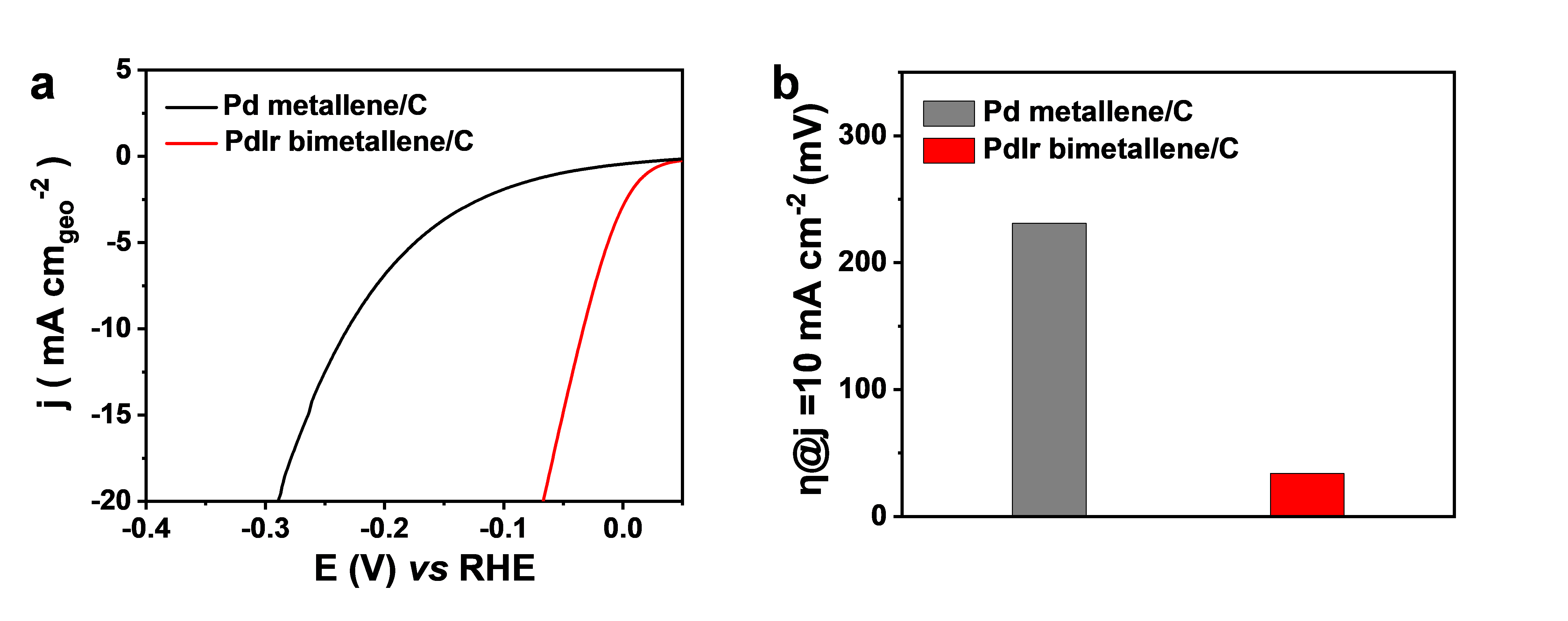


**Figure S16.** (a) HER polarization curves of PdIr bimetallene/C and Pd metallene/C at the scan rate of 5 mV s^-1^ with 95% iR-compensation. (b) Their overpotentials at current density of 10 mA cm^-2^.


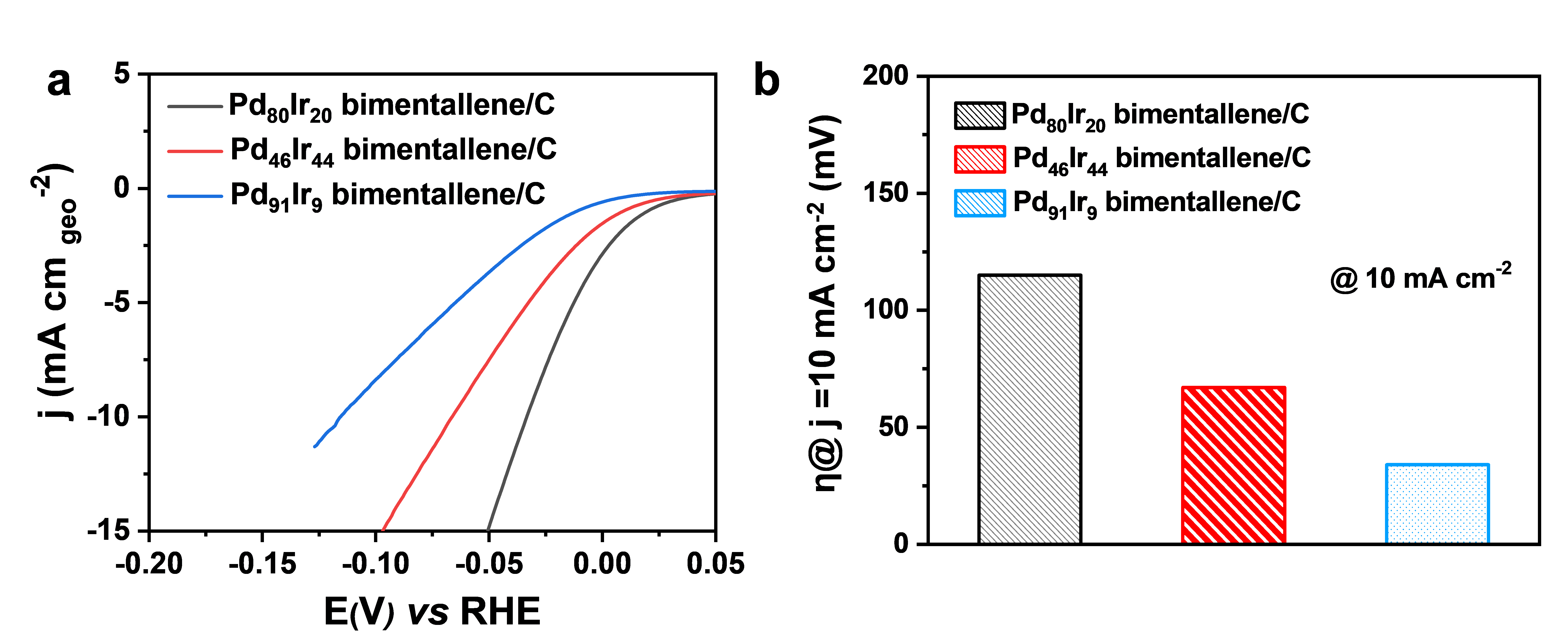


**Figure S17.** (a) The HER polarization curves of PdIr bimetallene with different Pd/Ir radio and (b) their overpotential at current density of 10 mA cm^-2^.

**Figure S18.** Specific activity of PdIr bimetallene/C, Pt/C, PdIr NP/C and Pd/C for HER at the potential of -70 mV *vs* RHE in 0.1 M KOH with 95% iR correction.

**Figure S19.** HER activities of various catalysts at an overpotential of 70 mV *vs.* RHE.

**Figure S20.** Chronoamperometric curves of the Pt/C catalysts at an applied potential of -0.02 and -0.07 V.


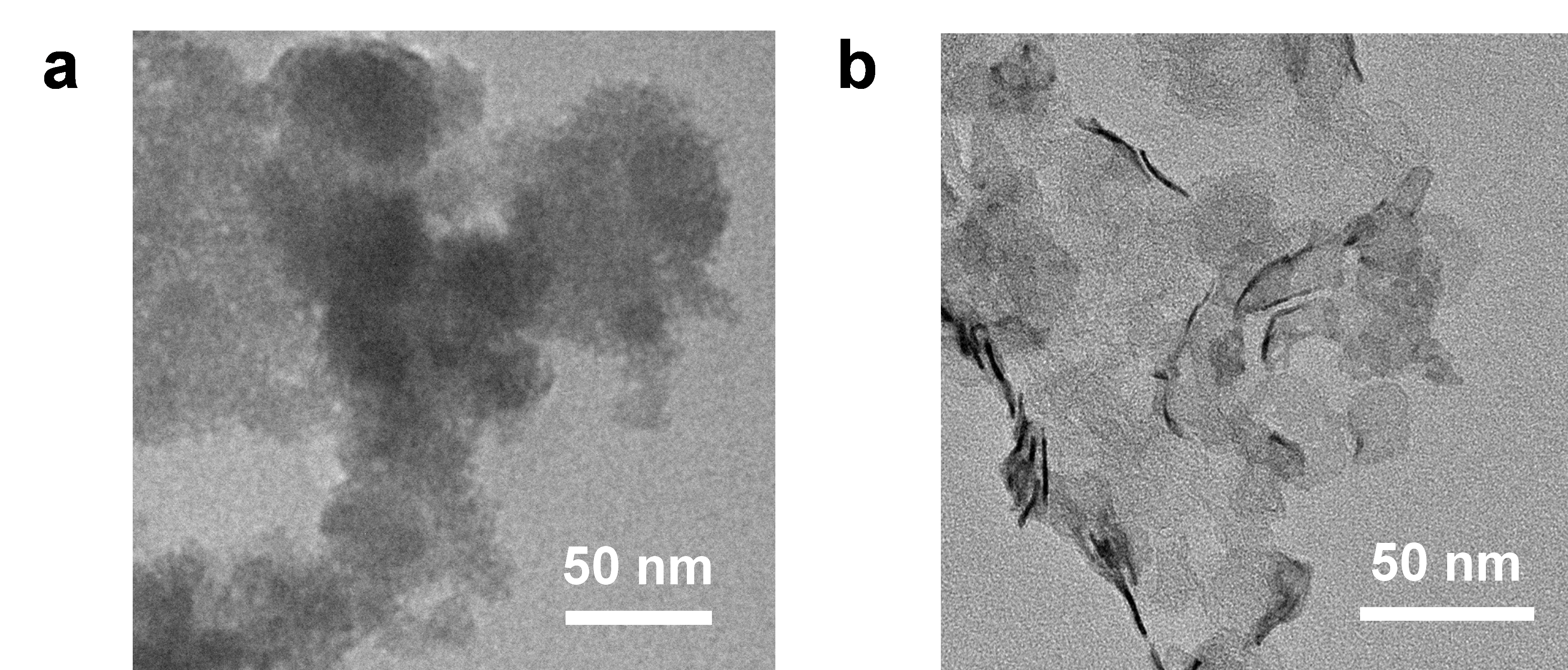


**Figure S21.** TEM images of Pt/C and PdIr bimetallene/C after HER stability test.


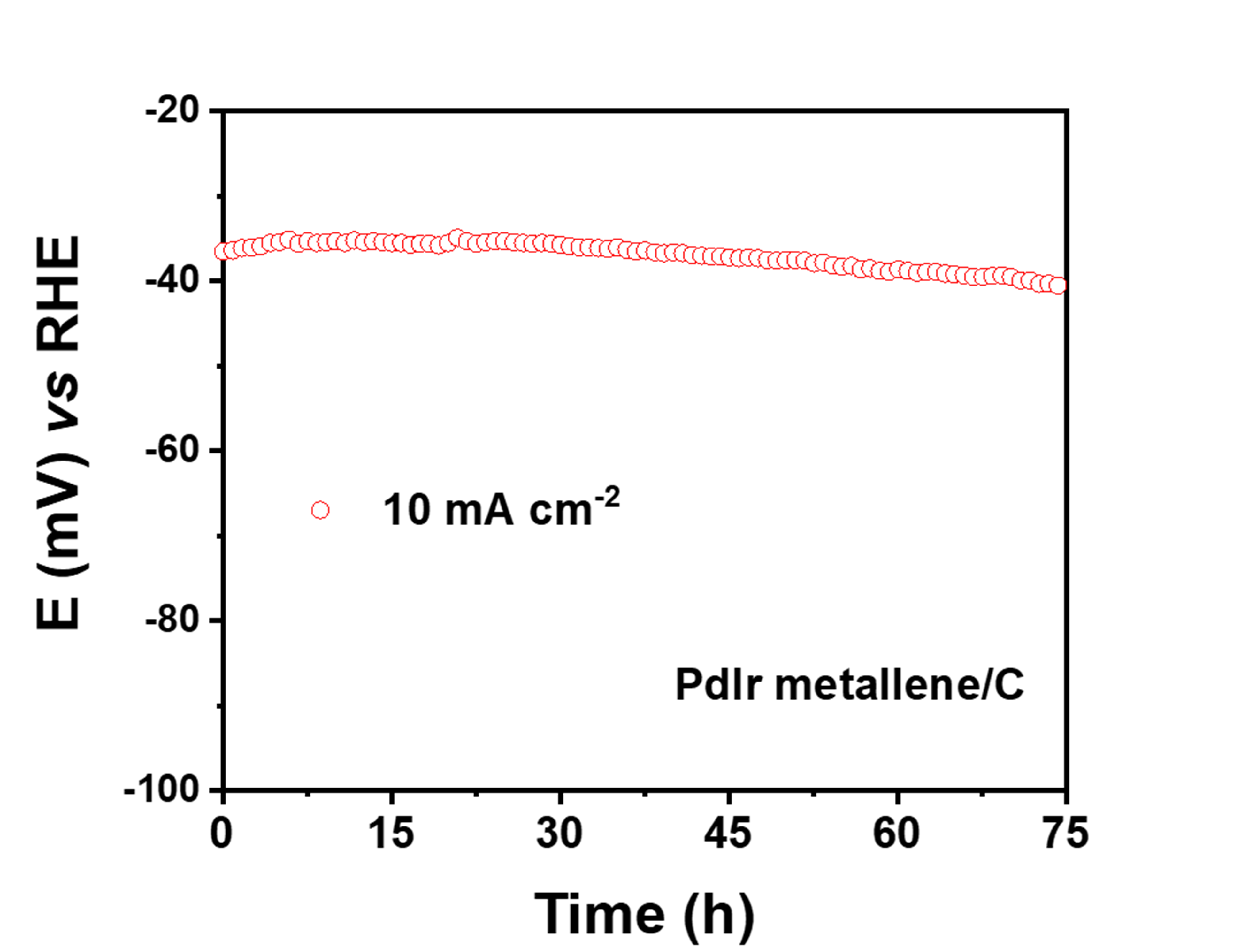


**Figure S22.** The chronopotentiometry measurement at 10 mA cm^-2^ of PdIr bimetallene at the mass loading of 0.2 mg_Pd+Ir_ cm^-2^ on CP.

**
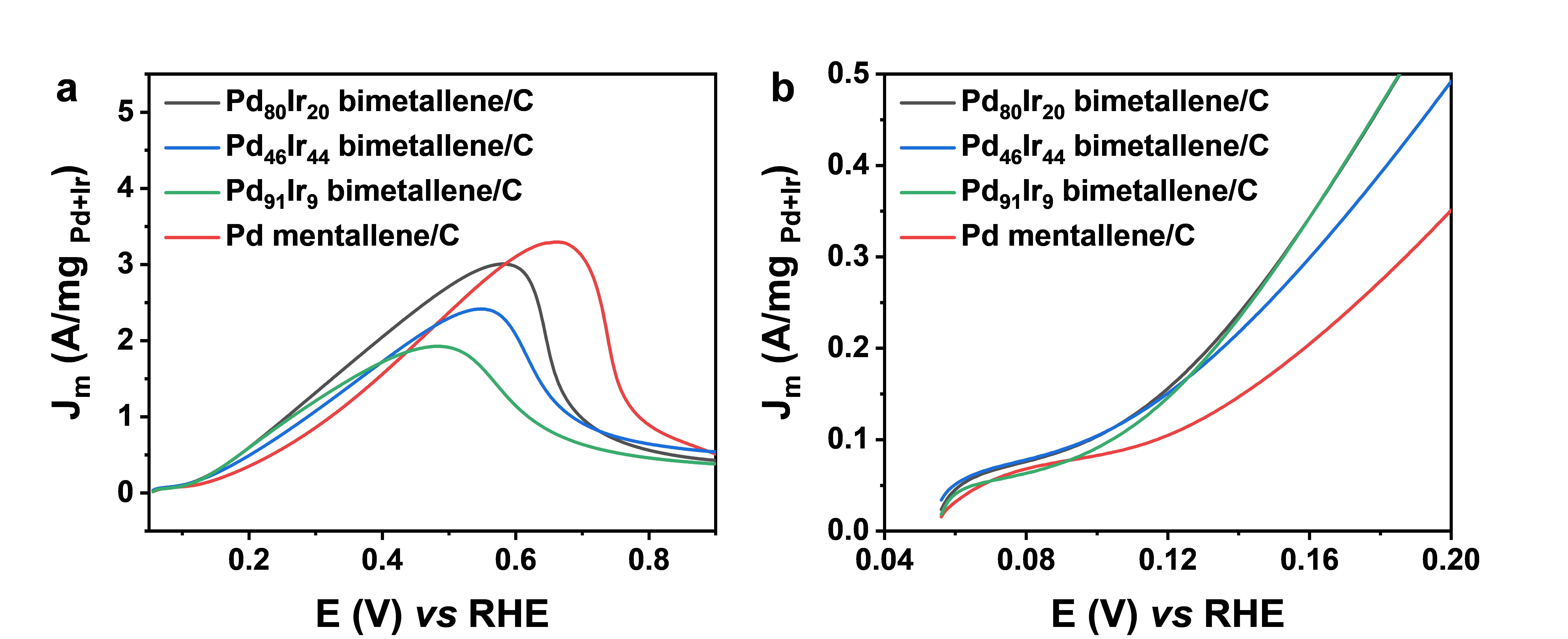
**

**Figure S23.** (a) CVs and (b) their onset potential of PdIr bimetallene with different Pd/Ir ratio, and Pd metallene for FAOR in 0.1 M HClO_4_ solution containing 0.5 M HCOOH at scan rate of 50 mV s^-1^.


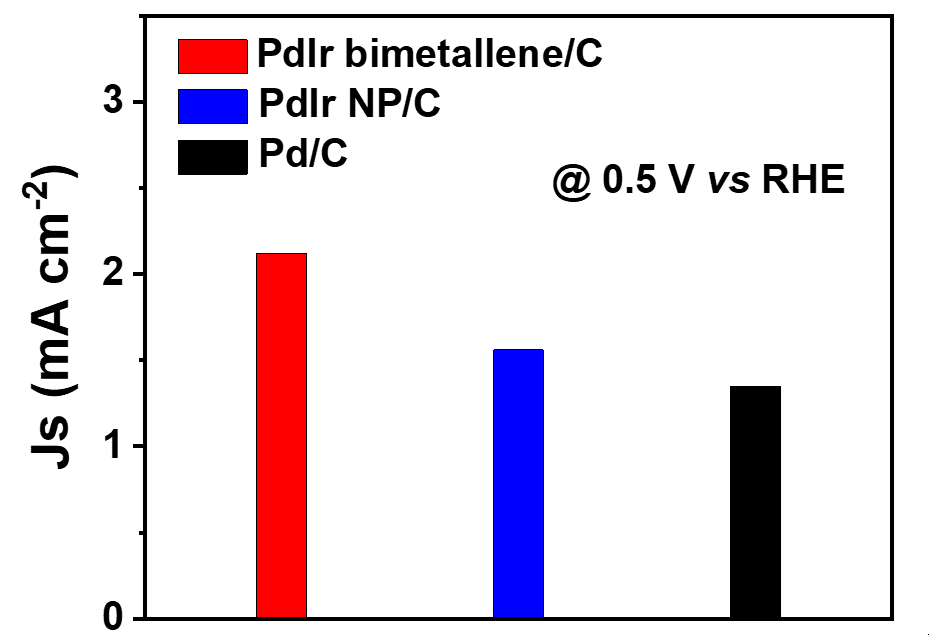


**Figure S24.** Specific activities of PdIr bimetallene/C, PdIr NP/C and Pd/C for FAOR at the potential of 0.5 V *vs.* RHE in 0.1 M HClO_4_ solution containing 0.5 M HCOOH.


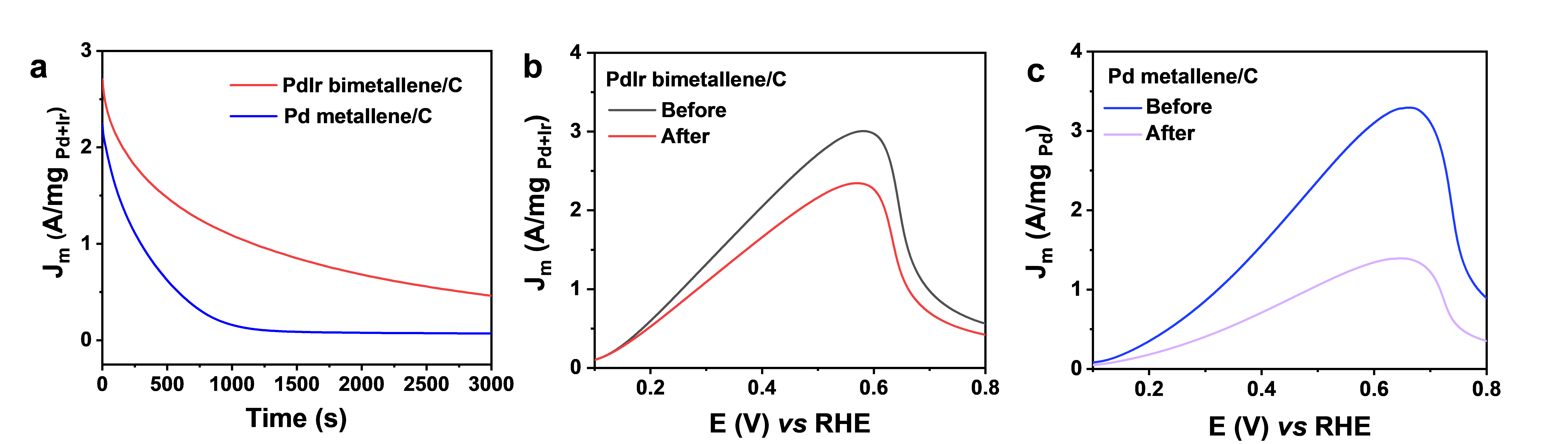


**Figure S25.** (a) The chronoamperometry of PdIr bimetallene/C and Pd metallene/C at 0.5 V *vs.* RHE in 0.1M HClO_4_ solution containing 0.5 M HCOOH and (b, c) their mass activities before and after chronoamperometry test.

**Figure S26.** The mass activities of PdIr NP/C and Pd/C for FAOR before and after chronoamperometry test.


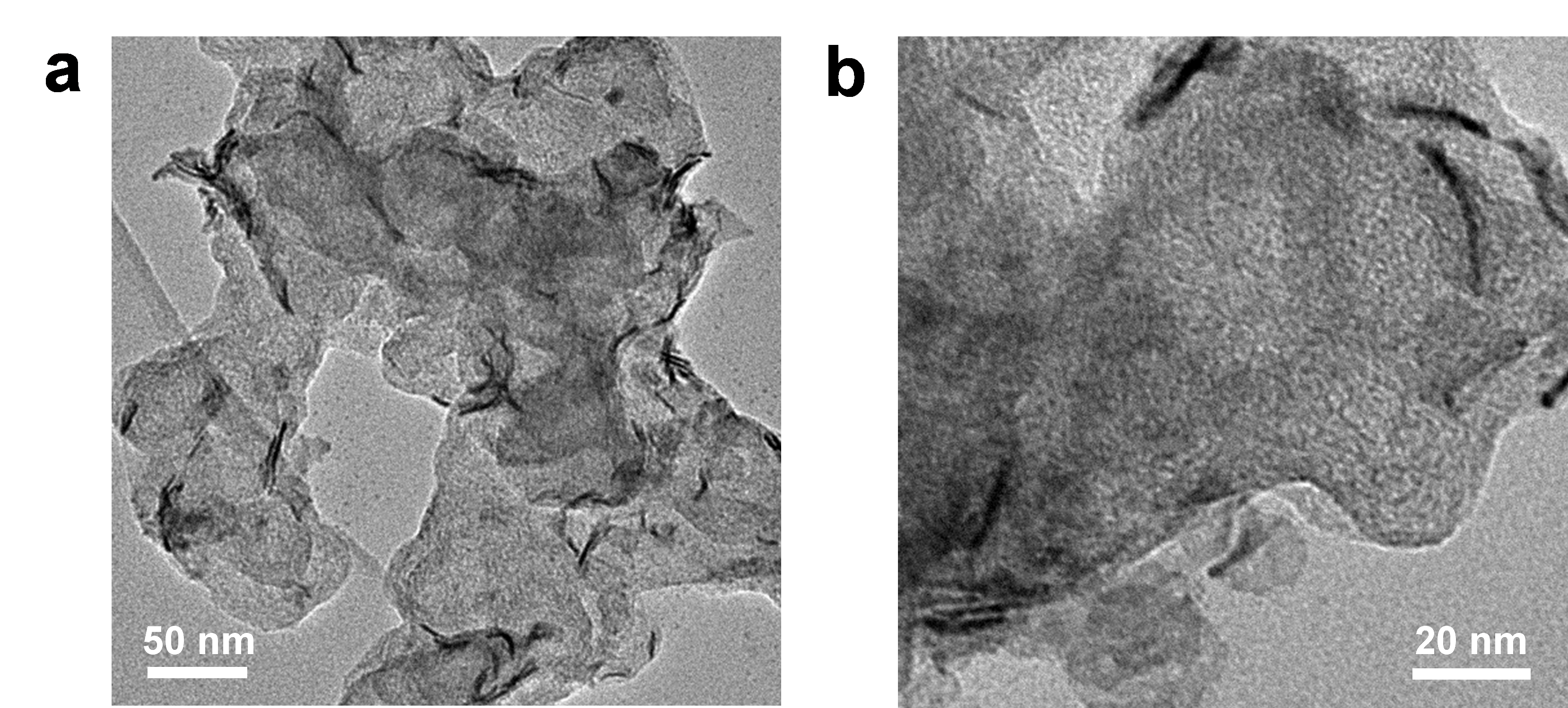


**Figure S27.** (a, b) TEM images of PdIr bimetallene at different magnification after FAOR for 3000 s.

**Figure S28.** The evolutions of the local structural configurations for the simulated FAOR process from the Pd_7_Ir_2_ (111) under acidic condition.

**Supplementary tables**

**Table S1.** Structural parameters of PdIr samples and standard materials extracted from the EXAFS fitting (^a^So^2^ = 0.78).

| **Samples** | **Scattering Pair** | **^b^CN** | **^c^R (Å)** | **^d^ơ^2^(10^-3^Å^2^)** | **^e^ΔE_0_(eV)** | **^f^R Factor** |
| --- | --- | --- | --- | --- | --- | --- |
| Pd foil | Pd-Pd | 12 | 2.74 | 5.18±0.19 | 3.48±0.22 | 0.0011 |
| PdIr metallene | Pd-Pd(Ir) | 7.1±1.3 | 2.77 | 12.1±0.19 | 9.64±0.86 | 0.012 |
| PdIr NP | Pd-Pd(Ir) | 8.5±1.2 | 2.74 | 5.64±0.16 | 5.64±0.96 | 0.013 |

^a^So^2^ is the amplitude reduction factor (obtained by the fitting of Pd foil); ^b^CN is the coordination number; ^c^R is interatomic distance (the bond length between Pd central atoms and surrounding coordination atoms); ^d^σ^2^ is Debye-Waller factor (a measure of thermal and static disorder in absorber-scatterer distances); ^e^ΔE_0_ is edge-energy shift (the difference between the zero kinetic energy value of the sample and that of the theoretical model). ^f^R factor is used to value the goodness of the fitting.

**Table S2.** The ECSA of various catalysts calculated from the charge of H_upd_ and CO stripping.

| **Samples** | **ECSA (H_upd_ m^2^/g_Pd and Ir_)** | **ECSA (CO stripping m^2^/g_Pd and Ir_ )** |
| --- | --- | --- |
| PdIr bimetallene/C | 130.3±9.2 | 127.5±10.8. |
| PdIr NPs/C | 38.9±5.0 | 42.8±3.2 |
| commercial Pt/C | 70.1±4.3 | / |
| commercial Pd/C | 38.6±2.8 | 40.5±3.0 |

**Table S3.** Summary of the ECSA values of the recently reported Pd-based nanocatalysts.

| **Catalyst** | **Morphology** | **ECSA(m^2^/g)** | **Reference** |
| --- | --- | --- | --- |
| PdIr | Nanosheet | 127.5±10.8 (PdIr) | This work |
| Pd | Porous Nanosheets | 12.9 (Pd) | *Adv. Funct. Mater.* 27, 1603852 (2017) |
| Pd | Nanomesh | 76.1 (Pd) | *Angew. Chem.* 130, 3493 (2018) |
| Pd | Nanosheet | 35.6 (Pd) | *Nano Lett.* 15, 7519 (2015) |
| Pd | Nanosheet | 67.0 (Pd) | *Nat. Nanotech.* 6, 28 (2011) |
| Pd | tetrapod | 15.9 (Pd) | *J. Am. Chem. Soc.* 134, 7073 (2012) |
| PdAg | 2D Nanodendrites | 69.5 (Pd) | *Adv. Mater.* 1706962 (2018) |
| PdCu | Nanosheet | 126.2± 12.5 (PdCu) | *Adv. Mater.* 29, 1700769 (2017) |
| PdCuCo | Anisotropic structure | 72.2 (Pd) | *Adv. Mater.* 30, 1704171 (2018) |
| PdCuCo | Nanodendrite | 58.7 (Pd) | *Nat. Comm.* 9, 3702 (2018) |
| Pd | Mesoporous Nanotube Arrays | 82.7 (Pd) | *Adv. Energy Mater.* 9, 1900955 (2019) |

**Table S4.** Summary of some recently reported HER electrocatalysts in alkaline electrolytes. *The values were roughly calculated from the data figures.

| **Sample** | **Mass Loading** | **Electrolyte** | **Current**  **Density (mA**  **cm^-2^)** | **Overpotential (mV)** | **Mass activity mA/ug (@ 50 mV)** | **Mass activity mA/ug (@ 70 mV)** | **Reference** |
| --- | --- | --- | --- | --- | --- | --- | --- |
| PdIr bimetallene/C | 10 μg cm^-2^ | 0.1 M KOH | 10 | 34 | 1.88 (Pd)  1.45 (PdIr) | 2.67 (Pd)  2.06 (PdIr) | This work |
| RhPd-H bimetallene | 15.3 µg cm^-2^ | 1 M KOH | 10 | 35.7 | / | / | *J. Am. Chem. Soc.* 142, 3645 (2020) |
| Au–Ru NWs | 80 ug cm^-2^ | 0.1 M KOH | 10 | 50 | ~0.59* (Ru)  ~0.125* (AuRu) | / | *Nat. Chem.* 10, 456 (2018) |
| Ru@C_2_N | 0.285 mg cm^–2^ | 1 M KOH | 10 | 17 | ~0.37* (Ru) | */* | *Nat. Nano.* 12, 441 (2017) |
| RuCo/C | 0.275 mg cm^–2^ | 1 M KOH | 10 | 28 | ~1.24* (Ru) | */* | *Nat. Comm.*  8, 14969 (2017) |
| RuCo/C | 0.153 mg cm^-2^ | 1 M KOH | 10 | 13 | ~0.53*  (Ru) | */* | *Nat. Comm.* 8, 4958 (2018). |
| Pt NWs/SL Ni(OH)_2_ | 16 μg cm^-2^ | 0.1 M KOH | 10 | 48 | */* | 1.59 (Pt) | *Nat. Common.* 6, 6430 (2015) |
| np-CuTi | - | 0.1 M KOH | 10 | 47 | / | / | *Nat. Commun.* 6, 6567 (2015) |
| NiO/Ni  -CNT | 0.28  mg cm^‑2^ | 1 M KOH | 10 | 80 | / | 0.025*(Ni) | *Nat. Commun.* 5, 4695 (2014) |
| Pt_3_Ni_2_-NWs-S/C | 15 μg cm^-2^ | 0.1 M KOH | 10 | 45 | / | 1.34 (Pt) | *Nat Commun.* 8, 14580 (2017) |

**Table S5.** Summary of the mass activities on Pd-based catalysts towards FAOR.

| **Catalyst** | **Test Condition** | **Mass Activity @ peak**  **(A/mg )** | **Reference** |
| --- | --- | --- | --- |
| PdIr bimetallene/C | 0.1 M HClO_4_ + 0.5 M FA  Scan rate : 50mV s^-1^ | 3.01 (PdIr)  3.76 (Pd) | This work |
| Pd  Nanosheet | 0.5 M H_2_SO_4_ + 0.5 M FA  Scan rate : 50 mV s^-1^ | 1.38 (Pd) | *Nat. Nanotech*. 6, 28 (2011) |
| Porous Pd nanosheet | 0.5 M H_2_SO_4_ + 0.5 M FA  Scan rate : 50 mV s^-1^ | 0.41 (Pd) | *Adv. Funct. Mater. 27*, 1603852 (2017) |
| Pd  Nanosheet | 0.1 M HClO_4_ + 0.2 M FA  Scan rate : 50 mV˖s^-1^ | 0.63 (Pd) | *Nano Lett.* 15, 7519-7525 (2015) |
| Pd concave  tetrahedron | 0.1 M HClO_4_ + 0.2 M FA  Scan rate : 50 mV˖s^-1^ | 0.46 (Pd) |
| Pd tetrahedron | 0.1 M HClO_4_ + 0.2 M FA  Scan rate : 50 mV˖s^-1^ | 0.24 (Pd) |
| PdCu Nanosheet | 0.5 M H_2_SO_4_ + 0.25 M FA  Scan rate : 50 mV˖s^-1^ | 1.63(Pd) | *Adv. Mater.* 29, 1700769 (2017) |
| PdCu  Nanotripods | 0.5 M HClO_4_ + 0.5 M FA  Scan rate : 50 mV˖s^-1^ | 1.58 (Pd) | *Adv. Funct. Mater.* 24, 7520 (2015) |
| PdCo NPs | 0.1 M HClO_4_ + 2 M FA  Scan rate:50 mV˖s^-1^ | 0.77 (Pd) | *Nano Lett.* 12, 1102 (2012). |
| Pd/CoP  Nanoparticle | 0.5 M H_2_SO_4_ + 0.5 M FA  Scan rate : 50 mV s^-1^ | 2.76 (Pd) | *Nano Energy* 30,355 (2016). |
| Palladium Nanotube Arrays | 0.5 m H_2_SO_4_ + 0.5 M FA  Scan rate:50 mV˖s^-1^ | 3.65(Pd) | *Adv. Energy Mater.* 9, 1900955 (2019) |
| Pd/PdWCr Nanoflowers | 0.5 m H_2_SO_4_ + 0.5 M FA  Scan rate:50 mV˖s-1 | 2.087(Pd) | *Adv. Funct. Mater.* 30, 2003933(2020) |
